# Predictive and prognostic markers from endoscopic ultrasound with biopsies during definitive chemoradiation therapy in esophageal squamous cell carcinoma

**DOI:** 10.1186/s12885-023-10803-8

**Published:** 2023-07-20

**Authors:** Qingwu Du, Xiaoyue Wu, Kunning Zhang, Fuliang Cao, Gang Zhao, Xiaoying Wei, Zhoubo Guo, Yang Li, Jie Dong, Tian Zhang, Wencheng Zhang, Ping Wang, Xi Chen, Qingsong Pang

**Affiliations:** 1 Departments of Radiation Oncology, Key Laboratory of Cancer Prevention and Therapy, Tianjin Medical University Cancer Institute and Hospital, National Clinical Research Center for Cancer, Tianjin’s Clinical Research Center for Cancer, Tianjin, China; 2 Departments of Endoscopy Diagnosis and Therapy, Key Laboratory of Cancer Prevention and Therapy, Tianjin Medical University Cancer Institute and Hospital, National Clinical Research Center for Cancer, Tianjin’s Clinical Research Center for Cancer, Tianjin, China; 3 Departments of Pathology, Key Laboratory of Cancer Prevention and Therapy, Tianjin Medical University Cancer Institute and Hospital, National Clinical Research Center for Cancer, Tianjin’s Clinical Research Center for Cancer, Tianjin, China; 4 Department of Nutrition Therapy, Key Laboratory of Cancer Prevention and Therapy, Tianjin Medical University Cancer Institute and Hospital, National Clinical Research Center for Cancer, Tianjin’s Clinical Research Center for Cancer, Tianjin, China

**Keywords:** Chemoradiation, Esophageal cancer, Interim response, Nomogram, Endoscopic ultrasound

## Abstract

**Introduction:**

Endoscopic ultrasound (EUS) may play a role in evaluating treatment response after definitive chemoradiation therapy (dCRT) for esophageal squamous cell carcinoma (ESCC). This study explored the prognostic markers of EUS with biopsies and developed two nomograms for survival prediction.

**Methods:**

A total of 821 patients newly diagnosed with ESCC between January 2015 and December 2019 were reviewed. We investigated the prognostic value of the changes in tumor imaging characteristics and histopathological markers by an interim response evaluation, including presence of stenosis, ulceration, tumor length, tumor thickness, lumen involvement, and tumor remission. Independent prognostic factors of progression-free survival (PFS) and overall survival (OS) were determined using Cox regression analysis and further selected to build two nomogram models for survival prediction. The receiver operating characteristic (ROC) curve, calibration curve, and decision curve analysis (DCA) were used to respectively assess its discriminatory capacity, predictive accuracy, and clinical usefulness.

**Results:**

A total of 155 patients were enrolled in this study and divided into the training (109 cases) and testing (46 cases) cohorts. Tumor length, residual tumor thickness, reduction in tumor thickness, lumen involvement, and excellent remission (ER) of spatial luminal involvement in ESCC (ER/SLI) differed significantly between responders and non-responders. For patients undergoing dCRT, tumor stage (P = 0.001, 0.002), tumor length (P = 0.013, 0.008), > 0.36 reduction in tumor thickness (P = 0.004, 0.004) and ER/SLI (P = 0.041, 0.031) were independent prognostic markers for both PFS and OS. Time-dependent ROC curves, calibration curves, and DCA indicated that the predicted survival rates of our two established nomogram models were highly accurate.

**Conclusion:**

Our nomogram showed high accuracy in predicting PFS and OS for ESCC after dCRT. External validation and complementation of other biomarkers are needed in further studies.

**Supplementary Information:**

The online version contains supplementary material available at 10.1186/s12885-023-10803-8.

## Introduction

Definitive chemoradiation therapy (dCRT) is a superior alternative for patients with unresectable esophageal cancer (EC) or those unwilling to undergo operative management [1]. In China, the pathological type of EC is, in the majority, esophageal squamous cell carcinoma (ESCC), which is dramatically inconsistent with the adenocarcinomas in Western countries [2]. In all, 40–75% of patients with ESCC may develop local recurrence after dCRT, with an increased risk of poor survival [3]. It has been reported that the clinical outcomes of EC significantly correlate with the tumor response to dCRT. Patients with a complete response (CR) to dCRT appear to be more likely to develop long-term survival, while those with residual disease are recommended to continue with reinforced or salvage treatment [4]. As a result, patients with less sensitivity to treatment are recommended to receive 60 Gy as a definitive radiation dose to improve localized control, rather than the international recommended standard dose of 50 Gy [5–8]. It is essential to evaluate the tumor response to dCRT, especially at an early timepoint, to predict the prognosis of patients with ESCC and determine whether additional reinforced therapeutic strategies are required.

Numerous studies have been carried out to evaluate the essential role of endoscopic ultrasound (EUS) in staging primary EC and treating early-stage EC with minimal local involvement [9]. EUS evaluations have been suggested to play a role in predicting pathological CR in patients with EC after neoadjuvant chemoradiation therapy (nCRT), and tumor thickness was significantly correlated with tumor remission grade. To date, relatively few studies have investigated the potential role of EUS in assessing tumor treatment response and predicting the prognosis of patients with ESCC who receive dCRT [10, 11].

At our institution, patients who underwent dCRT and had not received treatment previously for EC were recommended to have an interim response evaluation. EUS with biopsies was conducted when the radiation dose reached 40 Gy. Additional information was obtained about the tumor response to dCRT, which would greatly help guide subsequent management and assess prognosis [12, 13].

The current study aimed to evaluate the role of interim response evaluation in predicting treatment response and patient survival for ESCC according to a comparison of the results of pre- and interim-treatment EUS-based measurements.

## Materials and methods

### Patients and treatments

Between January 1, 2015, and December 31, 2019, a cohort of 821 patients who underwent dCRT and had not received treatment previously for EC (stage I-IVA) at the Tianjin Medical University Cancer Institute and Hospital were enrolled. Only biopsy-confirmed squamous cell carcinoma with pre- and interim-treatment EUS measurements was eligible for inclusion (Supplementary Figure S1). The included patients were categorized into training and testing cohorts (7:3) according to the timing of treatment initiation. The tumor-node-metastasis (TNM) staging system proposed by the American Joint Committee on Cancer (8th edition) was used. In patients who received dCRT, radiotherapy was performed with conventional fractionation schedules (5 days/week, 1.8–2.0 Gy/daily fraction). The radiation dose ranged from 45.0 to 63.0 Gy, with a median dose of 54.0 Gy, in 30 fractions using intensity modulated radiotherapy (IMRT) with concomitant chemotherapy, mainly 4 to 6 cycles (docetaxel and cisplatin, weekly).

### Treatment evaluation

**Fig. 1:**
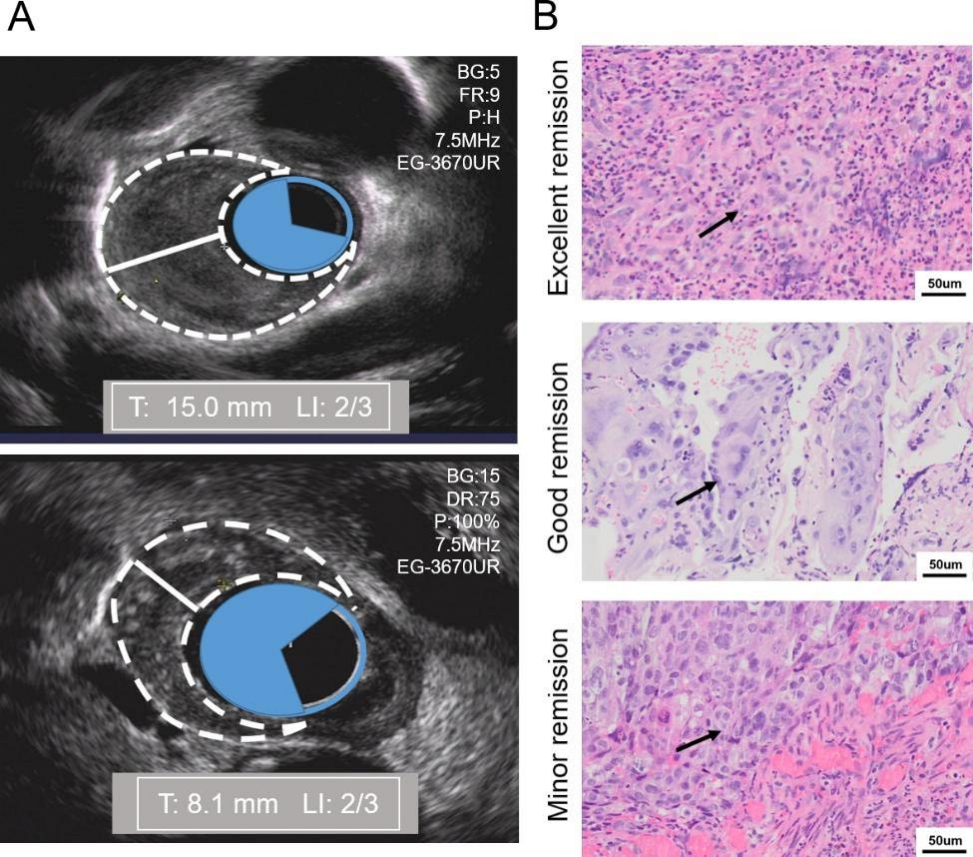
Tumor thickness, lumen involvement and histopathologic response of esophageal squamous cell carcinoma tissues during dCRT. (A) EUS-based evaluation of tumor thickness (white line) and lumen involvement (blue shaded area) pre- and interim-treatment in the same patient. (B) Tumor remission during dCRT was graded in 3 levels: excellent remission, good remission, and minor remission (arrows indicate residual tumor). Abbreviations: T, tumor thickness; LI, lumen involvement; dCRT, definitive chemoradiation therapy; EUS, endoscopic ultrasound

EUS examinations with biopsies were performed in patients with EC before dCRT treatment. When the radiation dose reached 40 Gy, usually four to five weeks into treatment, EUS with biopsies was repeated. At least three different sites of any suspicious lesions in the esophagus were considered for biopsies. Examination records were reviewed for the presence of stenosis, ulceration, tumor length, tumor thickness, lumen involvement, and tumor remission. Among them, the tumor length was the distance between the upper and lower edges of the esophageal tumor. The tumor thickness was measured in millimeters from the esophageal lumen to the outer tumor edge (Fig. 1A). In cases of an impassable esophagus, the maximum tumor thickness of the passable part was measured instead. Lumen involvement was defined as the proportion of spartial and circumferential tumors involving the esophageal lumen (Fig. 1A). The proportion of lumen involvement ≤ 0.67 during the interim evaluation was recorded as spatial luminal involvement (SLI). Histopathological evaluation was based on a scoring system originally designed to evaluate microscopic response to chemoradiation therapy (CRT) in ESCC [12, 14, 15]. Tumor remission was classified into three grades, and details are given in Fig. 1B and Supplementary Table S1. All patients were assessed clinically based on CT, esophagography, and ultrasonography of the neck and abdomen 1 month after dCRT, according to the Response Evaluation Criteria for Solid Tumors (RECIST 1.1) [16].

### Follow-up

Patients were monitored approximately every 3 months for 1 year, every 6 months for the next 2 years, and annually thereafter. Overall survival (OS) was measured from the initiation of treatment to the date of death or the date of the last follow-up. Progression-free survival (PFS) was calculated from the initiation of treatment to the date of lesion progression, including the primary tumor, regional lymph nodes, and distant areas. Locoregional recurrence (LRR) was defined as the time from the initiation of therapy to the progression of the irradiated regions. Patients with a LRR ≥ 24 months were classified as the group of responders [17].

### Statistical analyses

Continuous variables were analyzed using Mann-Whitney U test, and categorical variables were tested using the chi-square test or the Fisher exact test. Age, tumor length, baseline tumor thickness, residual tumor thickness, and lumen involvement were grouped with the median value. The cutoff value of the reduction in tumor thickness was calculated by the Youden index. Survival curves were plotted by the Kaplan–Meier method and assessed using the log-rank test. Univariate and multivariate Cox regression analyses were performed to generate hazard ratios (HR) and 95% confidence intervals (CI), and to explore the prognostic variables for constructing nomograms. The “pec,” “rms,” and “ggDCA” packages of R were applied to generate time-dependent receiver operating characteristic (ROC) curves, calibration curves, and decision curve analysis (DCA) for validating the nomogram, respectively [18]. All statistical tests were 2-tailed. A P < 0.05 represented a significant value. All analyses and data visualization were performed in SPSS (Version 25.0) and the R programming language (version 4.0.5).

## Results

### Patient characteristics and overall treatment response

A total of 155 patients were enrolled in this study, with a median age of 62 years (range, 44 to 82 years), and were divided into training (109 patients) and testing cohorts (46 patients). The median follow-up was 43.4 months (95% CI, 41.3 to 45.4 months). By December 2021, fifty (32.3%) patients were still alive and 119 (76.8%) patients experienced relapse, of which 62 (52.1%) suffered a local recurrence (LR), 45 (37.8%) suffered a regional recurrence (RR), and 35 (29.4%) suffered distant metastases (DMs). The 1-, 2-, and 3-year OS rates were 73.4%, 48.1%, and 36.1%, respectively. Fifty-nine (42.8%) patients were classified as active responders, and 79 (57.2%) patients as non-responders.

### Interim analysis

**Fig. 2:**
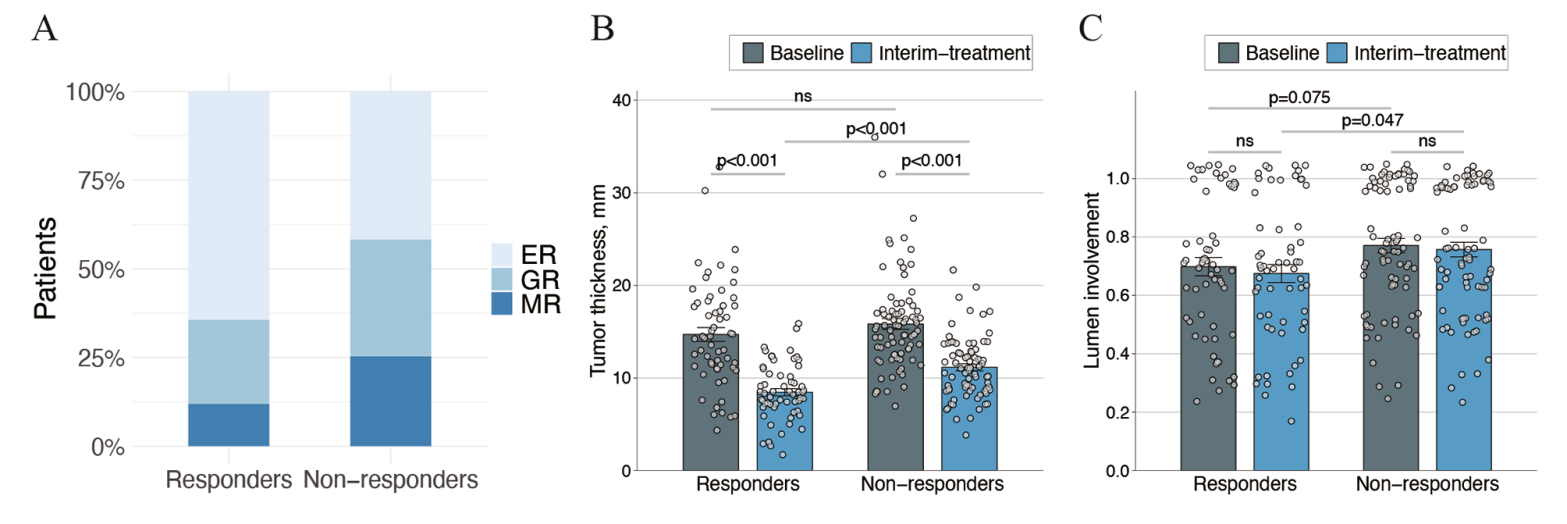
Distributions of tumor remission (A), tumor thickness (B) and lumen involvement (C) at baseline and during treatment between responders and non-responders

The presence of stenosis and ulceration were observed in 63 (40.6%) of 155 and 30 (19.4%) of 155 patients, respectively. Excellent remission (ER), good remission (GR), and minor remission (MR) were achieved by 77 (49.7%), 47 (30.3%), and 31 (20.0%) patients, respectively (Fig. 2A). The interim response evaluation revealed that, after CRT, tumor thickness was significantly reduced in patients with ESCC (P < 0.001) (Fig. 2B). However, no obvious changes of lumen involvement were observed during the treatment in these patients (P = 0.072) (Fig. 2 C).

### Prognostic significance of the interim analysis

A significant correlation was found between the interim response evaluation outcomes and responders. Stenosis (P = 0.043), tumor length (P < 0.001), residual tumor thickness (P < 0.001), reduction of tumor thickness (P < 0.001), and tumor remission (P = 0.024) were significantly different between responders and non-responders (Table 1). No significant correlations were found for ulceration (P = 0.399), baseline tumor thickness (P = 0.363) and lumen involvement at baseline (P = 0.057) and during treatment (P = 0.052) (Table 1). Responders had a lower proportion of lumen involvement than non-responders; however, the p-value was not significant. We then combined tumor remission with spatial luminal involvement (SLI) during treatment; ER and SLI tumors achieved higher predictive values for responders, with a specificity of 83.6% (66/79, 95%CI 73.1–90.6%, P < 0.001) (Table 1). Tumor clinical characteristics of responders and non-responders are shown in Supplementary Table S2.

**Table 1:** Interim analysis outcomes by EUS of responders and non-responders^†^

Variables	Responders (%)	Non-responders (%)	P
Stenosis			**0.043**
Yes	19 (32.2)	39 (49.4)	
No	40 (67.8)	40 (50.6)	
Ulceration			0.399
Yes	10 (16.9)	18 (22.8)	
No	49 (83.1)	61 (77.2)	
Tumor length, cm			**< 0.001**
≤ 5.0	43 (72.9)	31 (39.2)	
> 5.0	16 (27.1)	48 (60.8)	
Tumor thickness, mm			
Baseline < 15	30 (50.8)	34 (43.0)	0.363
≥ 15	29 (49.2)	45 (57.0)	
Residual < 10	41 (69.5)	30 (38.0)	**< 0.001**
≥ 10	18 (30.5)	49 (62.0)	
Reduction < 0.36	20 (33.9)	54 (68.4)	**< 0.001**
≥ 0.36	39 (66.1)	25 (31.6)	
Lumen involvement			
Baseline > 0.67	27 (45.8)	49 (62.0)	0.057
≤ 0.67	32 (54.2)	30 (38.0)	
Residual > 0.67	23 (39.0)	44 (55.7)	0.052
≤ 0.67	36 (61.0)	35 (44.3)	
Tumor remission			**0.024**
Minor	7 (11.9)	20 (25.3)	
Good	14 (23.7)	26 (32.9)	
Excellent	38 (64.4)	33 (41.8)	
Combination analysis			**< 0.001**
ER and SLI	29 (49.2)	13 (16.5)	
Others	30 (50.8)	66 (83.5)	

^†^138 patients were included in the analysis; the remaining 17 patients were not included due to the short follow-up time. Abbreviations: ER, excellent remission; SLI, spatial luminal involvement.

### Independent prognostic factors for PFS and OS

**Fig. 3:**
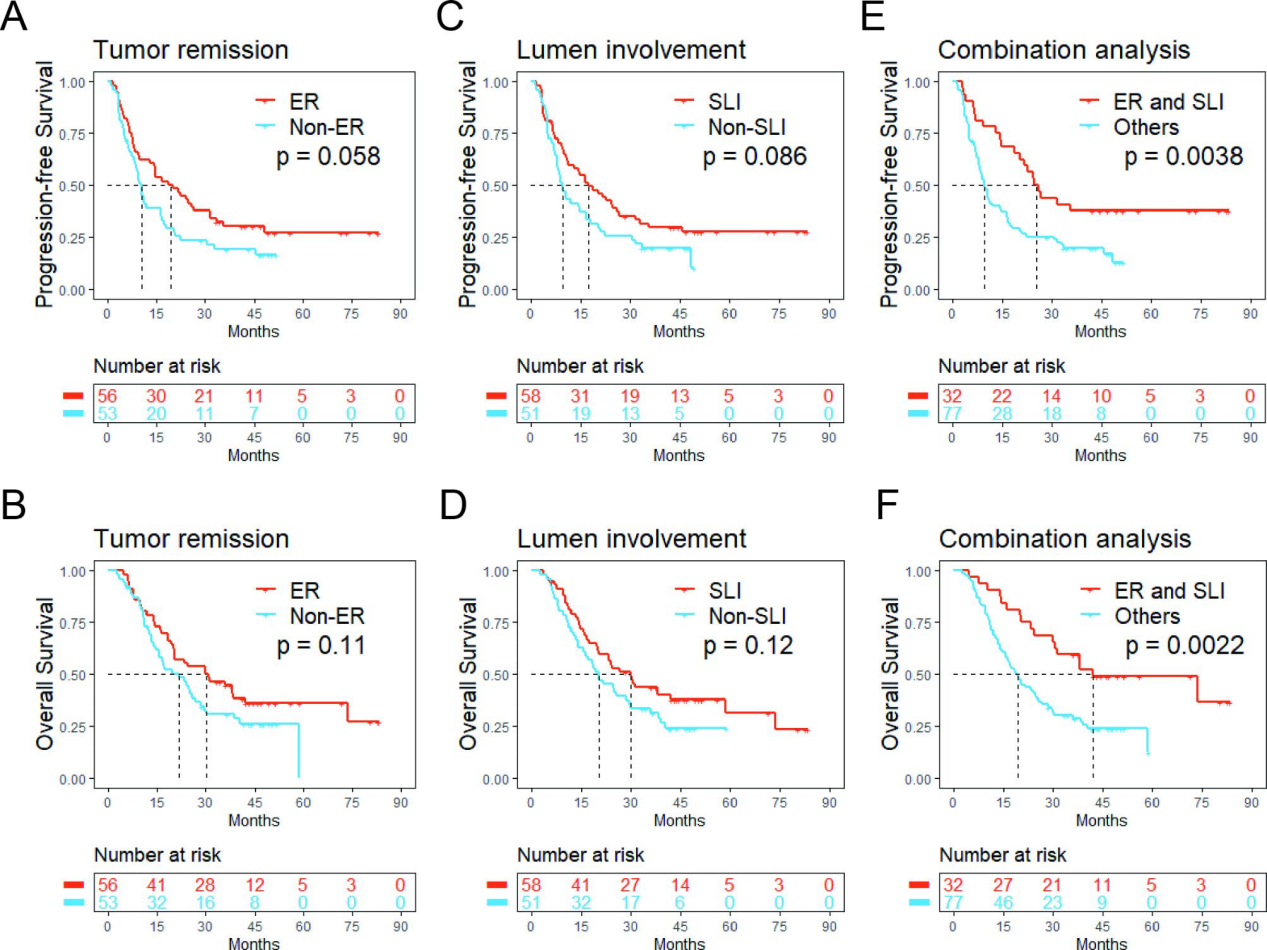
Kaplan–Meier curves depicting the differing impact of tumor remission and lumen involvement on PFS and OS for patients treated with dCRT. (A, B) tumor remission; (C, D) lumen involvement; (E, F) combination analysis. Abbreviations: ER, excellent remission; SLI, spatial luminal involvement

Survival data for the primary cohort were analyzed. Patients with ER or SLI tended to have improved PFS and OS compared to those without, although the difference was not significant (PFS, P = 0.058, 0.086; OS, P = 0.11, 0.12). However, patients with both ER and SLI had significantly better PFS and OS than those with ER alone, SLI alone, or neither (PFS, P = 0.004; OS, P = 0.002) (Fig. 3).

Table 2 shows the results of the univariate Cox regression analysis. Stenosis, baseline T, baseline N, TNM stage, tumor length, and reduction in tumor thickness were associated with PFS and OS. Residual tumor thickness was only associated with OS (P = 0.042), but not with PFS (P = 0.109). Since the TNM stage combines the depth of tumor infiltration and the status of lymph node metastasis, we only included the TNM stage for further multivariate Cox regression analysis. Finally, TNM stage (P = 0.001, 0.002), tumor length (P = 0.013, 0.008), reduction in tumor thickness (P = 0.004, 0.004), and ER/SLI (P = 0.041, P = 0.031) were found to be independent prognostic factors for PFS and OS (Table 3).

**Table 2:** Patient and tumor characteristics and univariate Cox regression of variables associated with PFS and OS

Variables	N (%)	PFS			OS	
		HR (95% CI)	P value		HR (95% CI)	P value
Age,median,y	62(44–82)	0.875(0.568–1.346)	0.543		1.042(0.662–1.642)	0.857
Male sex	90(82.6)	1.502(0.830–2.717)	0.178		1.366(0.735–2.536)	0.324
Smoke	79(72.5)	1.083(0.787–1.491)	0.625		0.998(0.694–1.437)	0.992
T1-3 vs. T4	85(78.0)	1.822(1.099–3.022)	**0.020**		1.864(1.114–3.119)	**0.018**
N0-1 vs. N2-3	71(65.1)	1.813(1.163–2.826)	**0.009**		2.005(1.262–3.186)	**0.003**
TNM category			**0.002**			**0.003**
I/II	23(21.1)	1.0			1.0	
III	55(50.5)	1.758(0.958–3.225)	0.069		2.237(1.114–4.491)	0.024
IV	31(28.4)	3.156(1.643–6.064)	0.001		3.552(1.704–7.406)	0.001
Ulceration	20(18.3)	1.203(0.697–2.076)	0.507		1.172(0.654-2.100)	0.593
Stenosis	42(38.5)	1.594(1.029–2.467)	**0.037**		1.640(1.036–2.596)	**0.035**
Tumor length > 5.0 cm	50(45.9)	1.622(1.051–2.502)	**0.029**		1.769(1.122–2.789)	**0.014**
Tumor thickness (mm)						
Baseline ≥ 15 mm	52(47.7)	0.999(0.649–1.538)	0.997		1.117(0.709–1.760)	0.633
Residual ≥ 10 mm	49(45.0)	1.426(0.924–2.201)	0.109		1.608(1.017–2.543)	**0.042**
Reduction ≥ 0.36	51(46.8)	0.557(0.359–0.865)	**0.009**		0.548(0.345–0.872)	**0.011**
lm involvement						
Baseline > 0.67	55(50.5)	1.307(0.848–2.013)	0.225		1.264(0.803–1.990)	0.312
Residual > 0.67	51(46.8)	1.458(0.946–2.248)	0.088		1.437(0.909–2.273)	0.121
Tumor remission			0.123			0.230
Minor	20(18.3)	1.0			1.0	
Good	30(27.5)	0.577(0.333-1.000)	0.050		0.616(0.344–1.103)	0.103
Excellent	59(54.1)	0.795(0.434–1.454)	0.456		0.829(0.440–1.563)	0.562
Combination analysis						
ER and SLI	32(29.4)	1.0			1.0	
others	77(70.6)	0.480(0.289–0.798)	**0.005**		0.428(0.245–0.748)	**0.003**

Abbreviations: PFS, progression free survival; OS, overall survival; HR, hazard ratios; CI, confidence intervals; ER, excellent remission; SLI, spatial luminal involvement.

**Table 3:** Multivariable Cox regression of variables associated with PFS and OS

Variables	PFS			OS	
	HR (95% CI)	P		HR (95% CI)	P
Stenosis	1.508(0.964–2.359)	0.072		1.528(0.948–2.463)	0.081
TNM category		**0.001**			**0.002**
I/II	1.0			1.0	
III	1.124(0.596–2.121)	0.717		1.504(0.728–3.106)	0.271
IV	3.009(1.530–5.918)	0.001		3.588(1.646–7.821)	0.001
Tumor length	1.770(1.128–2.780)	**0.013**		1.903(1.185–3.057)	**0.008**
Tumor thickness					
Residual > 10 mm				0.838(0.478–1.470)	0.538
Reduction > 0.36	0.491(0.301-0.800)	**0.004**		0.456(0.265–0.782)	**0.004**
Others vs. ER and SLI	0.576(0.339–0.977)	**0.041**		0.479(0.245–0.934)	**0.031**

Abbreviations: PFS, progression free survival; OS, overall survival; HR, hazard ratios; CI, confidence intervals; ER, excellent remission; SLI, spatial luminal involvement.

### Development and validation of nomograms

**Fig. 4:**
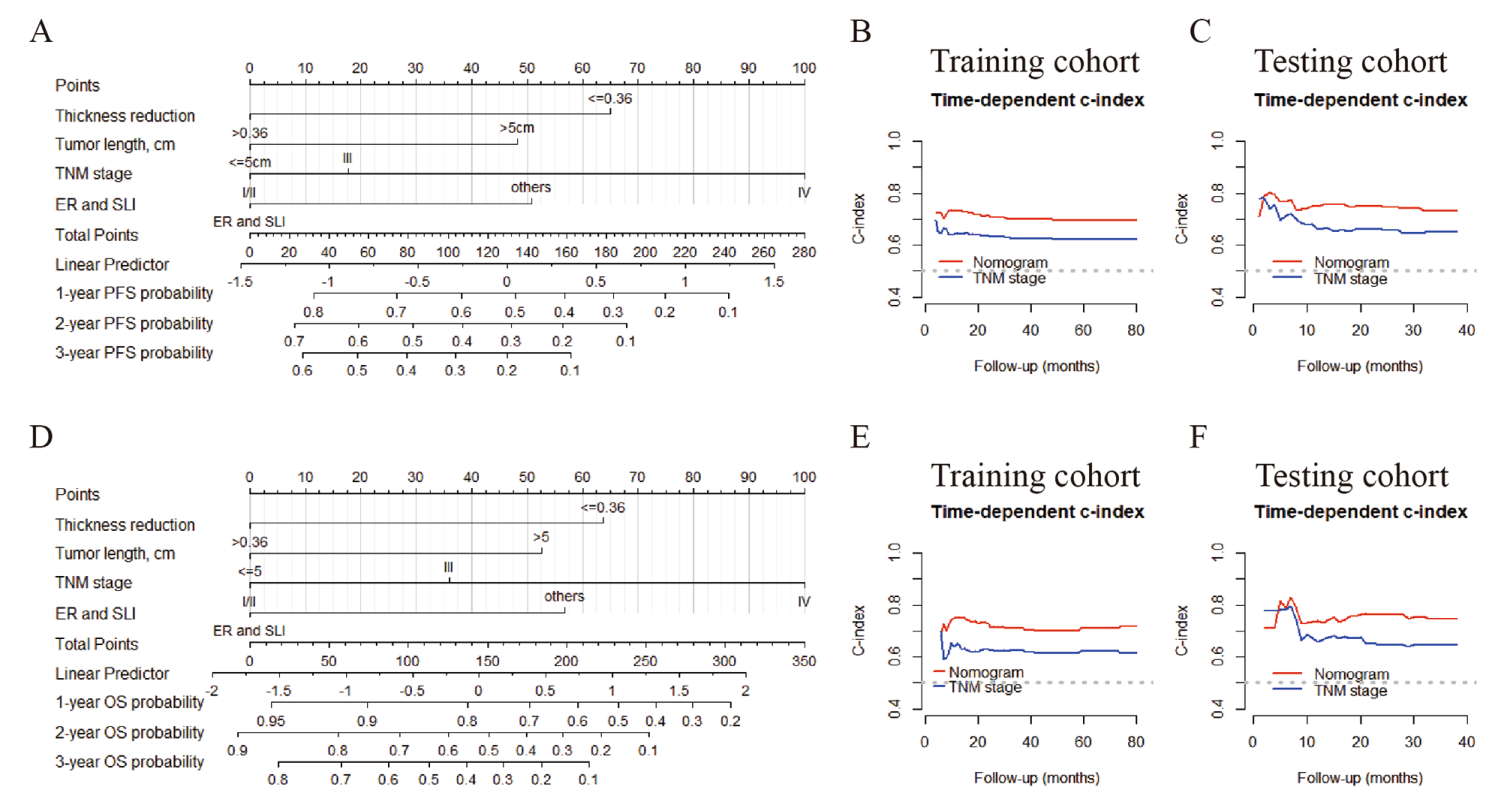
Construction and validation of the nomogram for predicting PFS and OS. The nomogram for predicting 1-, 2-, and 3-year PFS (A) and OS (D) of patients with ESCC. Time-dependent ROC curves of the nomogram for PFS (B, C) and OS (E, F) prediction in the training and the testing cohorts. Abbreviations: ER, excellent remission; SLI, spatial luminal involvement; PFS, progression-free survival; OS, overall survival

Using the four independently prognostic markers, we constructed two nomograms to predict the 1-, 2-, and 3-year PFS and OS for ESCC patients who received dCRT (Fig. 4A, D). The possibilities of PFS and OS at 1, 2 and 3 years were estimated by adding the points of each item on the nomogram.

We mainly evaluated the predictability of the nomograms from three aspects: discrimination, calibration, and clinical effectiveness. Our nomograms yielded a C-index of 0.713 (95% CI 0.663–0.762) in predicting PFS and 0.711 (95% CI 0.657–0.765) in predicting OS. Additionally, the time-dependent ROC curves of the nomograms demonstrated a better discrimination of PFS (Fig. 4B, C) and OS (Fig. 4E, F) than those of the TNM stage at almost all time points in the follow-up, in both the training and testing cohorts. The calibration curves and the DCA diagrams also indicated the superior accuracy of the predictions (Supplementary Figure S2).

## Discussion

In the current study, we assessed both tumor imaging characteristics and histopathological performance by EUS with biopsies to capture the changes in tumor burden from the macro and micro perspectives, which indicated the response of tumors to dCRT. We found that reduction in tumor thickness, tumor length, ER/SLI, and TNM stage were significantly associated with local control and the survival probability of patients with ESCC. Furthermore, we established two visualized nomogram models combining these prognostic markers, which may enable clinicians to stratify prognostic subgroups and adapt the therapeutic schedule for patients with ESCC.

Most studies try to assess the early response of tumors to dCRT in patients with EC using magnetic resonance imaging (MRI), positron emission tomography with integrated computed tomography (PET/CT), or other imaging examinations. Quantitative imaging values, especially for classical MRI parameters, such as ADC values, differ between scanners and might not be standardized, as the values of ADC vary in different MRI devices [19, 20]. Another nonnegligible disadvantage is that MRI or PET/CT cannot discern the lesions confined to the mucosal layer [17]. Sakurada et al. combined high-resolution T2-weighted images with diffusion-weighted magnetic resonance imaging (DWI) for the detection of early EC, and only 33% of T1 carcinomas were detected accurately [21]. Subsequently, some other studies have highlighted the value of EUS in overcoming these limitations, which had become a standard measurement to identify tumor histological types and assist in tumor staging of patients with EC.

The predictive value of EUS has been confirmed in the nCRT field. A single-center retrospective study revealed that tumor thickness reduced by more than 50% after neoadjuvant therapy was significantly associated with tumor downgrade and long-term survival [22]. Another substudy of the preSANO trial did not find an association between tumor response and the reduction ratio of tumor thickness [11]. However, an absolute value of tumor thickness > 4.5 mm after completion of nCRT could predict residual disease with a sensitivity of 87%. In our present study, both residual thickness and changes in tumor thickness showed a significant correlation with active responders (Residual, P < 0.001; reduction in thickness, P < 0.001). However, survival analysis showed that only changes in tumor thickness were correlated with patient survival rates and were considered as an independent prognostic marker of PFS and OS. The possible causes for this discrepancy may be the difference in tumor histological types and the varied cutoff values of tumor thickness in different studies.

Compared with esophageal adenocarcinoma, posttreatment endoscopic biopsy played an improved role in predicting treatment response to CRT of ESCC. Qian et al. found that ER combined with tumor-infiltrating lymphocytes > 60% (LPE, lymphocyte-predominant ESCC) during CRT correlated with pCR and cancer-specific survival for patients with ESCC. During the interim response evaluation, patients evaluated with ER/LPE tended to have more improved survival rates compared with other patients who had ESCC [12]. Similar results were found in our present study, patients with ER or SLI were associated with a better PFS and OS in ESCC; however, the difference was not significant. When we combined both factors, patients with ER/SLI obtained significantly improved PFS (P = 0.004) and OS (P = 0.002) rates than other patients. We have also found a significant correlation between ER/SLI and responders. ER/SLI showed high predictive values for 2-year locoregional control, with a specificity of 83.6%. In other words, cases that did not achieve both ER and LIE during treatment tended to have locoregional failure after dCRT. This information obtained at the early timepoint of the treatment would have great importance in guiding subsequent management and assessing prognosis.

To our knowledge, our study is the first to assess EUS-based measurements pre- and interim-dCRT to evaluate the tumor response to dCRT in ESCC patients. Compared with the individual value before or during treatment, we believed that the reduction in tumor thickness was more significant in tumor assessment clinically for patients with ESCC who received dCRT, which was also consistent with the findings from other previous studies [22–25]. Using changes in tumor thickness appears superior for early tumor response evaluation compared to downgrading traditional EUS-based T staging [24, 26]. Another strength of this study was the relatively complete clinical data, including both tumor imaging characteristics and histopathological evaluation at baseline and during the treatment, which helped to assess the tumor response to dCRT comprehensively. In addition, at least three different sites of any suspicious lesions were considered for biopsies. Similar to bite-on-bite biopsies in the preSANO trial [10], a second biopsy sample was conducted at the same site, which helped detect residual tumors. Finally, we integrated the clinical parameters and pathological markers of tumors and established two visualized nomogram models with high accuracy in predicting PFS and OS.

Many studies have built prognostic models to predict the treatment response and survival probability of patients with EC who receive CRT [20, 27–29]. However, few studies have been specifically designed for squamous histology, whose clinicopathological features and biological behavior remarkably differ from adenocarcinoma. Only patients with ESCC who had complete clinical data and evaluation records were included in our study to ensure the reliability and accuracy of our nomograms. In addition, our nomograms are the first EUS-based models that are well-validated in another relatively independent cohort of patients with ESCC who were treated with dCRT. The selected prognostic markers in our nomograms were available to clinicians and easily applied to clinical practice. With great predictive performance in both the training cohort and testing cohorts, we believed that our models would have great potential to guide clinicians in assessing the overall treatment response and adapting therapy strategies of patients with ESCC. Low-risk cases may have satisfactory long-term survival, so close surveillance is recommended. However, other cases with poor treatment response may require a multidisciplinary approach, and a more intensive radiation dose is suggested.

We acknowledge the limitations of our analysis. In the interim response evaluation of 29 impassable esophagi, we measured the tumor thickness of the passable part, which may be less than the actual maximum tumor thickness, resulting in a limited influence on the results. Second, it is the genome that determines the sensitivity of esophageal cancer to CRT; from this point of view, our nomograms may not adequately assess the true response. A nomogram based on CD8, Foxp3, CD33, and PD-L1 yielded an AUC of 0.764 for 5-year OS prediction [30]. Other molecular biomarkers, such as ALDH1 and GLI1/HH, which have been reported to be associated with the therapeutic response [31], could be of complementary value to develop the current models. Finally, this was a retrospective single-institution analysis, and the performance of our models need to be validated in a larger external cohort.

## Conclusion

Based on prognostic markers acquired from EUS with biopsies, we constructed and validated two individual nomograms to estimate PFS and OS in patients with ESCC who underwent dCRT. Before application in clinical practice, our current models must be validated and further refined, and their incorporation with other critical molecular biomarkers should also be further explored.

## Electronic supplementary material

Below is the link to the electronic supplementary material.

**Supplementary Materials**: **Supplementary Figure S1**: Patient selection. **Supplementary Figure S2**: Figure S2 Validation of the nomogram for predicting PFS and OS. (A-D) Calibration curves and DCA of the nomogram for PFS prediction in the training and the testing cohorts. (E-H) Calibration curves, and DCA of the nomogram for OS prediction in the training and the validation cohorts. Abbreviations: PFS, progression-free survival; OS, overall survival; DCA, decision curve analysis. **Supplementary Table S1**: The scoring grades of chemoradiotherapy effect†,^1-4^. **Supplementary Table S2**: Figure S2 Tumor Characteristics of responders and non-responders.

## Data Availability

The clinicopathological data used to support the findings of this study are available from the corresponding author upon request.

## List of abbreviations

dCRT: Definitive chemoradiation therapy
EC: Esophageal cancer
ESCC: Esophageal squamous cell carcinoma
CR: Complete response
EUS: Endoscopic ultrasound
nCRT: Neoadjuvant chemoradiation therapy
CRT: Chemoradiation therapy
OS: Overall survival
PFS: Progression-free survival
LRR: Locoregional recurrence
HR: Hazard ratios
CI: Confidence intervals
ROC: Receiver operating characteristic()
DCA: Decision curve analysis
ER: Excellent remission
GR: Good remission
MR: Minor remission
SLI: Spatial luminal involvement
MRI: Magnetic resonance imaging
PET/CT: Positron emission tomography with integrated computed tomography
DWI: Diffusion-weighted magnetic resonance imaging
LPE: Lymphocyte-predominant esophageal squamous cell carcinoma
