# Correction: Predictive and prognostic markers from endoscopic ultrasound with biopsies during definitive chemoradiation therapy in esophageal squamous cell carcinoma

**DOI:** 10.1186/s12885-023-11206-5

**Published:** 2023-07-25

**Authors:** Qingwu Du, Xiaoyue Wu, Kunning Zhang, Fuliang Cao, Gang Zhao, Xiaoying Wei, Zhoubo Guo, Yang Li, Jie Dong, Tian Zhang, Wencheng Zhang, Ping Wang, Xi Chen, Qingsong Pang

**Affiliations:** 1 Departments of Radiation Oncology, Key Laboratory of Cancer Prevention and Therapy, Tianjin Medical University Cancer Institute and Hospital, National Clinical Research Center for Cancer, Tianjin’s Clinical Research Center for Cancer, Tianjin, China; 2 Departments of Endoscopy Diagnosis and Therapy, Key Laboratory of Cancer Prevention and Therapy, Tianjin Medical University Cancer Institute and Hospital, National Clinical Research Center for Cancer, Tianjin’s Clinical Research Center for Cancer, Tianjin, China; 3 Departments of Pathology, Key Laboratory of Cancer Prevention and Therapy, Tianjin Medical University Cancer Institute and Hospital, National Clinical Research Center for Cancer, Tianjin’s Clinical Research Center for Cancer, Tianjin, China; 4 Department of Nutrition Therapy, Key Laboratory of Cancer Prevention and Therapy, Tianjin Medical University Cancer Institute and Hospital, National Clinical Research Center for Cancer, Tianjin’s Clinical Research Center for Cancer, Tianjin, China

**Correction: BMC Cancer 23, 681 (2023)**

**https://doi.org/10.1186/s12885-023-10803-8**

Following publication of the original article [1], the authors identified that the versions of Fig. 2 and Fig. 4 are incorrect. The figures published in this correction article are correct and the original article [1] has been corrected.

**Fig. 2:**
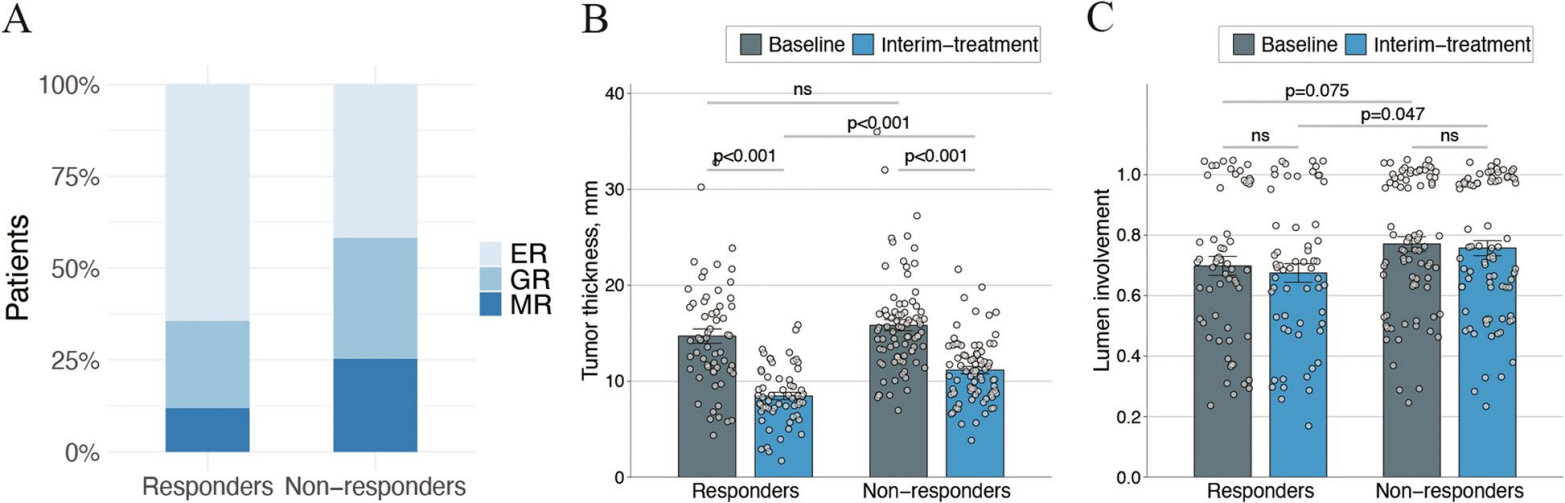
Distributions of tumor remission (**A**), tumor thickness (**B**) and lumen involvement (**C**) at baseline and during treatment between responders and non-responders

**Fig. 4:**
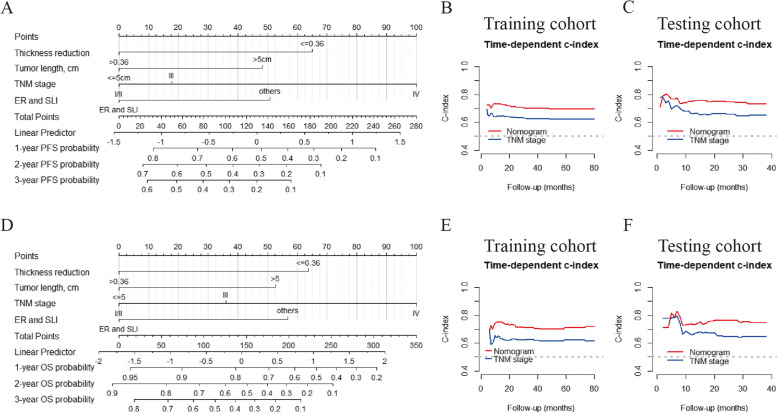
Construction and validation of the nomogram for predicting PFS and OS. The nomogram for predicting 1-, 2-, and 3-year PFS (**A**) and OS (**D**) of patients with ESCC. Time-dependent ROC curves of the nomogram for PFS (**B**, **C**) and OS (**E**, **F**) prediction in the training and the testing cohorts. Abbreviations: ER, excellent remission; SLI, spatial luminal involvement; PFS, progression-free survival; OS, overall survival
