# Supplementary material for: Predictive and prognostic markers from endoscopic ultrasound with biopsies during definitive chemoradiation therapy in esophageal squamous cell carcinoma
Source: BMC Cancer. 2023 Jul 20;23:681. doi: 10.1186/s12885-023-10803-8 (PMC10357763; doi:10.1186/s12885-023-10803-8)
Supplement: Supplementary file 1 — Supplementary Materials: Supplementary Figure S1: Patient selection. Supplementary Figure S2: Figure S2 Validation of the nomogram for predicting PFS and OS. (A-D) Calibration curves and DCA of the nomogram for PFS prediction in the training and the testing cohorts. (E-H) Calibration curves, and DCA of the nomogram for OS prediction in the training and the validation cohorts. Abbreviations: PFS, progression-free survival; OS, overall survival; DCA, decision curve analysis. Supplementary Table S1: The scoring grades of chemoradiotherapy effect†,1-4. Supplementary Table S2: Figure S2 Tumor Characteristics of responders and non-responders. [file 12885_2023_10803_MOESM1_ESM.docx]

**Supplementary Materials**

**Supplementary Figure S1**


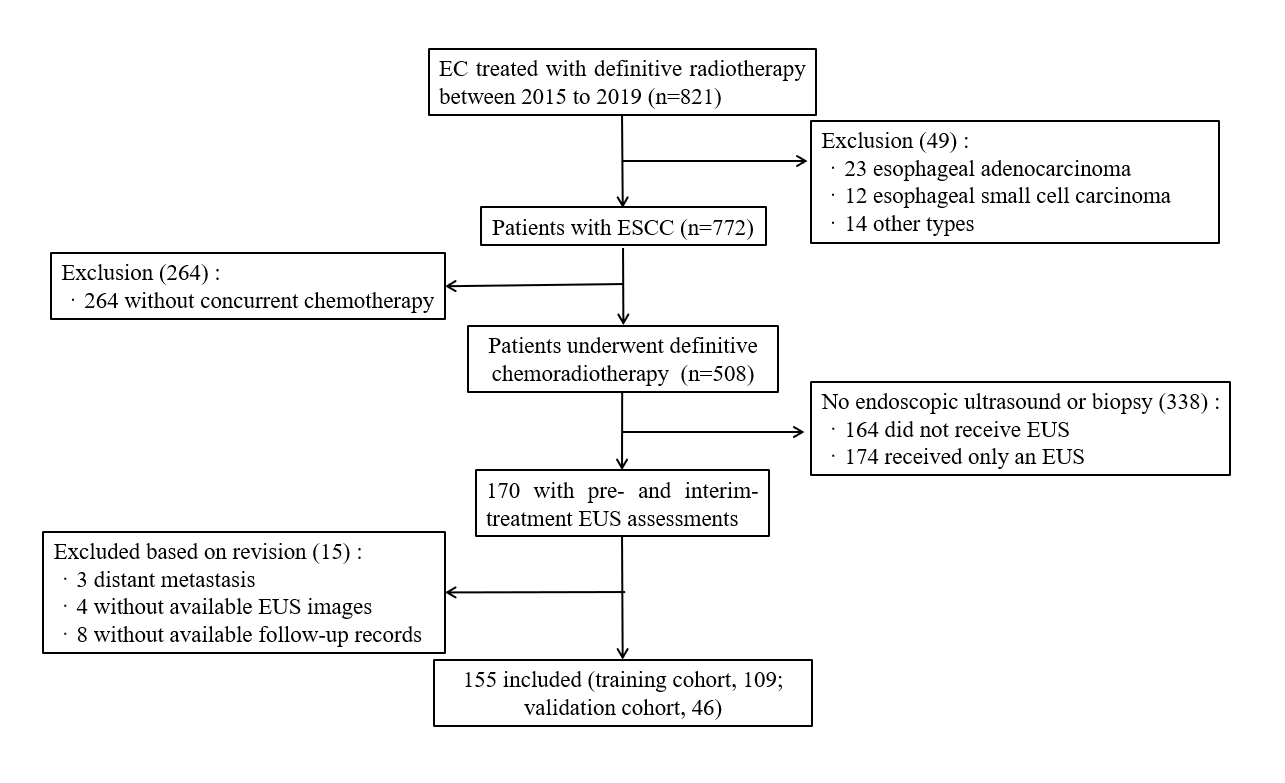


Patient selection.

**Supplementary Figure S2**

**
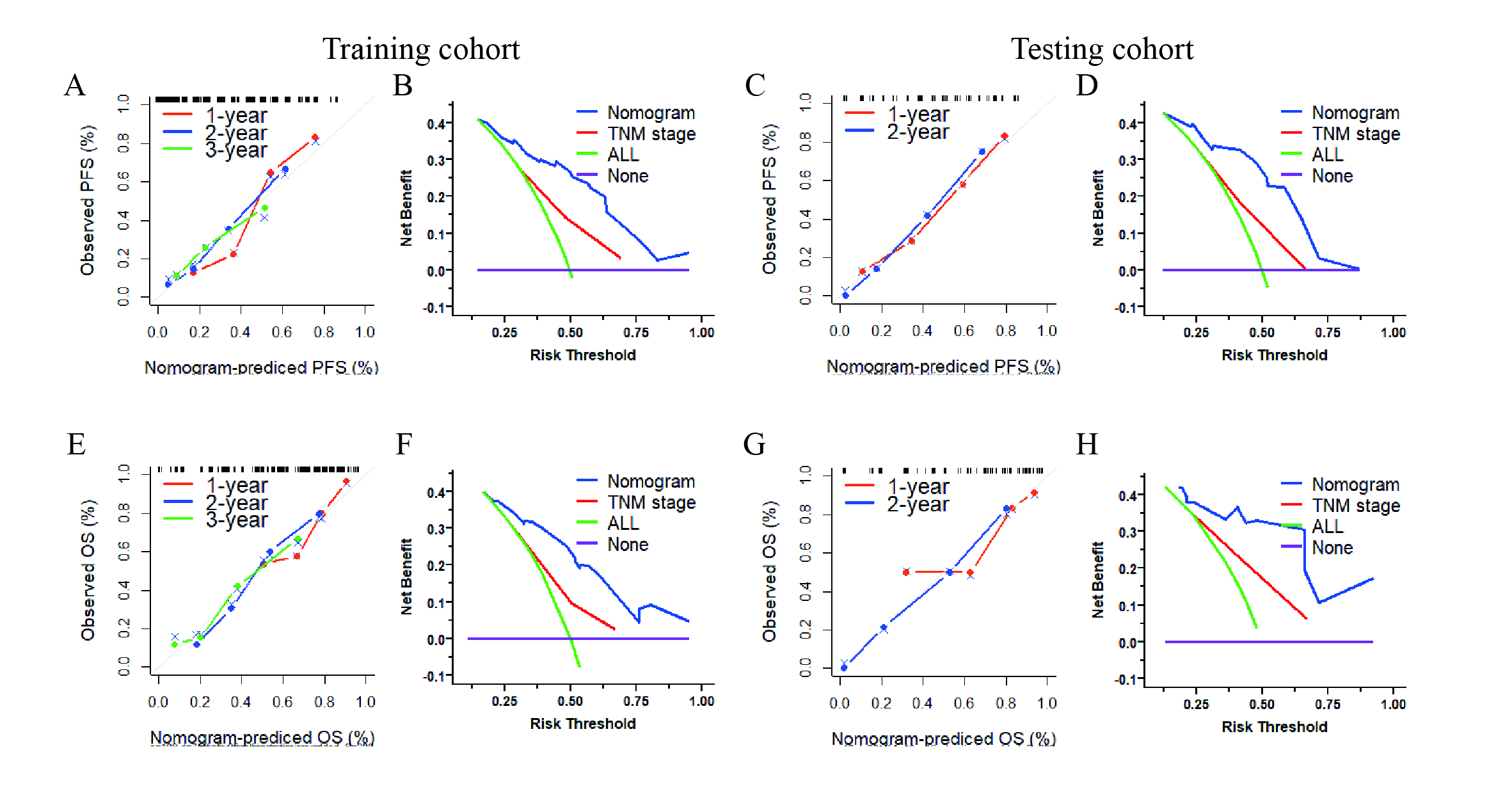
**

Figure S2 Validation of the nomogram for predicting PFS and OS. (A-D) Calibration curves and DCA of the nomogram for PFS prediction in the training and the testing cohorts. (E-H) Calibration curves, and DCA of the nomogram for OS prediction in the training and the validation cohorts. Abbreviations: PFS, progression-free survival; OS, overall survival; DCA, decision curve analysis.

**Supplementary Table S1**

The scoring grades of chemoradiotherapy effect^†,1-4^

| Rating |  | Effect |
| --- | --- | --- |
| Excellent | +++ | Destruction, negative mitosis and disappearance of nuclei; Severe degeneration of cytoplasm; Complete destruction of the cancer nestle. |
| Good | ++ | Negative mitosis; Pyknotic changes of nuclei; Vacuolization of the cytoplasm; moderate destruction and transformation of the cancer nestle. |
| Minor | +/- | Positive/negative mitosis; Basophilic change of cytoplasm; Polymorphic cells; Nuclei present; Proliferation of cancer nestle |

^†^Tumor remission during chemoradiotherapy when induction chemoradiation finished.

**Supplementary Table S1 Reference:**

1. Qian D, Wang Y, Zhao G, et al. Tumor Remission and Tumor-Infiltrating Lymphocytes During Chemoradiation Therapy: Predictive and Prognostic Markers in Locally Advanced Esophageal Squamous Cell Carcinoma. Int J Radiat Oncol Biol Phys 2019; 105: 319-328.

2. Yu JP, Lu WB, Wang JL, et al. Pathologic response during chemo-radiotherapy and variation of serum VEGF levels could predict effects of chemo-radiotherapy in patients with esophageal cancer. Asian Pac J Cancer Prev 2015; 16: 1111-1116.

3. Brun E, Zatterstrom U, Kjellen E, et al. Prognostic value of histopathological response to radiotherapy and microvessel density in oral squamous cell carcinomas. Acta Oncol 2001; 40: 491-496.

4. Akakura I, Nakamura Y, Kakegawa T, et al. Surgery of carcinoma of the esophagus with preoperative radiation. Chest. 1970 Jan;57(1):47-57.

**Supplementary Table S2**

Figure S2 Tumor Characteristics of responders and non-responders

| Variables | Responders (%) | Non-responders (%) | P |
| --- | --- | --- | --- |
| Location |  |  | 0.063 |
| Cervical | 14 (23.7) | 9 (11.4) |  |
| Upper thorax | 20 (33.9) | 19 (24.1) |  |
| Mid thorax | 20 (33.9) | 40 (50.6) |  |
| Lower thorax | 5 (8.5) | 11 (13.9) |  |
| Pathological differentiation |  |  |  |
| Poor | 26 (44.1) | 28 (35.4) | 0.499 |
| Good | 24 (40.7) | 34 (43.0) |  |
| Well | 9 (15.3) | 17 (21.5) |  |
| Shape of tumor |  |  | 0.296 |
| Protruding | 21 (35.6) | 24 (30.4) |  |
| Ulcerative | 15 (25.4) | 15 (19.0) |  |
| Combined | 17 (28.8) | 35 (44.3) |  |
| Superficial | 6 (10.2) | 5 (6.3) |  |
| T stage |  |  | 0.006 |
| T1-2 | 9 (15.3) | 2 (2.5) |  |
| T3 | 40 (67.8) | 51 (64.6) |  |
| T4 | 10 (16.9) | 26 (32.9) |  |
| N stage |  |  | 0.005 |
| N0 | 15 (25.4) | 6 (7.6) |  |
| N1 | 31 (52.5) | 37 (46.8) |  |
| N2 | 8 (13.6) | 25 (31.6) |  |
| N3 | 5 (8.5) | 11 (13.9) |  |
| TNM stage |  |  |  |
| I/II | 20 (33.9) | 6 (7.6) | <0.001 |
| III | 28 (47.5) | 42 (53.2) |  |
| IV | 11 (18.6) | 31 (39.2) |  |
| ^†^138 patients were included in the analysis of the training cohort; the remaining 17 patients were not included due to the short follow-up time. Abbreviations: ER, excellent remission; SLI, spatial luminal involvement. | | | |
